# Agreement between low-dose and ultra-low-dose chest CT for the diagnosis of viral pneumonia imaging patterns during the COVID-19 pandemic

**DOI:** 10.1186/s43055-021-00689-6

**Published:** 2022-01-05

**Authors:** Hooman Bahrami-Motlagh, Yashar Moharamzad, Golnaz Izadi Amoli, Sahar Abbasi, Alireza Abrishami, Mehdi Khazaei, Amir H. Davarpanah, Morteza Sanei Taheri

**Affiliations:** 1 Department of Radiology, School of Medicine, Shahid Beheshti University of Medical Sciences, Tehran, Iran; 2 Department of Radiology and Imaging Sciences, Emory University Hospital, Emory University School of Medicine, Atlanta, GA 30322 USA; 3 Department of Radiology, Shohada Hospital, Tajrish Sq., 1445613131 Tehran, Iran

**Keywords:** COVID-19, Computerized tomography, Ultra-low-dose, Chest

## Abstract

**Background:**

Chest CT scan has an important role in the diagnosis and management of COVID-19 infection. A major concern in radiologic assessment of the patients is the radiation dose. Research has been done to evaluate low-dose chest CT in the diagnosis of pulmonary lesions with promising findings. We decided to determine diagnostic performance of ultra-low-dose chest CT in comparison to low-dose CT for viral pneumonia during the COVID-19 pandemic.

**Results:**

167 patients underwent both low-dose and ultra-low-dose chest CT scans. Two radiologists blinded to the diagnosis independently examined ultra-low-dose chest CT scans for findings consistent with COVID-19 pneumonia. In case of any disagreement, a third senior radiologist made the final diagnosis. Agreement between two CT protocols regarding ground-glass opacity, consolidation, reticulation, and nodular infiltration were recorded. On low-dose chest CT, 44 patients had findings consistent with COVID-19 infection. Ultra-low-dose chest CT had sensitivity and specificity values of 100% and 98.4%, respectively for diagnosis of viral pneumonia. Two patients were falsely categorized to have pneumonia on ultra-low-dose CT scan. Positive predictive value and negative predictive value of ultra-low-dose CT scan were respectively 95.7% and 100%. There was good agreement between low-dose and ultra-low-dose methods (kappa = 0.97; *P* < 0.001). Perfect agreement between low-dose and ultra-low-dose scans was found regarding diagnosis of ground-glass opacity (kappa = 0.83, *P* < 0.001), consolidation (kappa = 0.88, *P* < 0.001), reticulation (kappa = 0.82, *P* < 0.001), and nodular infiltration (kappa = 0.87, *P* < 0.001).

**Conclusion:**

Ultra-low-dose chest CT scan is comparable to low-dose chest CT for detection of lung infiltration during the COVID-19 outbreak while maintaining less radiation dose. It can also be used instead of low-dose chest CT scan for patient triage in circumstances where rapid-abundant PCR tests are not available.

## Introduction

Chest CT scan has an important role in the diagnosis and management of COVID-19 infection caused by severe acute respiratory syndrome coronavirus 2 (SARS-CoV-2) [1]. In previous studies, chest CT was introduced as a highly sensitive method to screen for COVID-19 pneumonia [2]. A previous meta-analysis showed that chest CT scan has a pooled sensitivity of 94% and pooled specificity of 37% in the diagnosis of COVID-19 [3]. However, the studies included in this meta-analysis showed considerable heterogeneity that precludes conclusive results. The causes of heterogeneity include different methods of diagnosis confirmation (e.g., repeated RT-PCR test), difference in the prevalence of the infection reported from different geographic locations, and other variables.

Low-dose CT scan is a promising method shown to have acceptable diagnostic accuracy in the diagnosis of COVID-19 pneumonia [4–6]. In a previous study, low-dose chest CT was demonstrated to have a sensitivity of 86.7% and specificity of 93.6% for the diagnosis of COVID-19 [4].

Non-enhanced chest CT scan has been proposed as an option to assess the possibility of COVID-19 infection in adults [7]. Since radiation dose is a main concern, especially when managing asymptomatic individuals, efforts have been made to reduce the radiation dose [5, 8]. To the best of our knowledge, no study has used ultra-low-dose chest CT for this purpose.

Ultra-low-dose chest CT has been proven effective for screening lung nodules [9]. Additionally, ultra-low-dose CT was defined as an effective method to reduce radiation dose as well as motion artifact, and its radiation dose equals chest X-ray [10].

This prospective study was performed to determine diagnostic performance of ultra-low-dose chest CT compared to low-dose chest CT in detecting lung infiltration during the COVID-19 pandemic.

## Methods

### Study design

A total of 167 patients were prospectively enrolled in the current study. The patients were candidates for coronary angiography or other elective surgeries at a hospital in Tehran, Iran. The consecutive sample underwent chest CT scan two times. First, low-dose chest CT was performed. Then, ultra-low-dose CT was performed shortly after the low-dose scan. Low-dose chest CT scan is recommended by the Iranian Ministry of Health for the management of patients with suspected COVID-19 pneumonia [11].

### Chest CT protocols

Images were obtained with a single General Electric LightSpeed-4 scanner (GE, Milwaukee, WI, USA). Scanning parameters for low-dose and ultra-low-dose protocols are presented in Table 1. The volume of CT dose index (CTDI_vol_) was a fixed number for each protocol (Table 1). Dose-length product (DLP) was variable according to the thoracic length in each patient, and the numbers for low-dose and ultra-low-dose protocols were reported in the summary page of each CT scan. Effective dose (ED) was calculated by the formula of DLP × *k*, in which *k* [mSv/(mGy cm)] was set to 0.014 for chest CT according to a report from the American Association of Physicists in Medicine published in 2008 [12].

**Table 1:** Scan parameters of low-dose and ultra-low-dose chest CT protocols

Protocol	Low-dose	Ultra-low-dose
Scan type	Helical	Helical
Rotation time (s)	0.5	0.5
Beam collimation (mm)	40	40
Detector configuration	4 × 1.25 mm	4 × 1.25 mm
Pitch	1.5	1.5
Speed (mm/rot)	0.6	0.6
Tube voltage, kV	100	100
Tube current, mA	50	10
SFOV (scan field of view)	Large body	Large body
CTDIvol (CT dose index)	1.15	0.34

### Image analysis

Two radiologists (SA and GIA with six and five years of experience in reporting chest CT scan, respectively) independently reviewed all ultra-low-dose CT images first and decided on positive or negative CT findings suggestive of COVID-19 pneumonia, based on previous reports [13]. A third radiologist (HBM) checked the results, and in case of non-accordance, read-out was performed by a senior radiologist (MST) with 18 years of experience in reporting chest CT scan who was blinded to low-dose images. After one week, the above protocol was repeated for low-dose scans, and the results were recorded.

Chest CT findings for each protocol was recorded by a single radiologist (SA). Chest CT score was calculated based on visual estimation of involvement percent in each lobe as following: 0 for none, 1 for 1–25%, 2 for 26–50%, 3 for 51–75% and 4 for 76–100%. The sum of five lobe scores recorded as the total chest CT score (range between 0 and 20) [14].

### Sample size

A former study reported the sensitivity of low-dose chest CT to diagnose COVID-19 pneumonia as 86.7% [4]. Since we anticipated the sensitivity value of ultra-low-dose chest CT to be comparable with low-dose CT with a precision of 10% and prevalence of COVID-19 diagnosis about 30% at the sampling location, the minimum required sample size was calculated as 150 patients.

### Statistical analyses

Descriptive statistics, including frequency, percentage, mean, and standard deviation (SD), were used to describe the data. Diagnostic performance of ultra-low-dose chest CT was reported using sensitivity, specificity, positive predictive value (PPV), and negative predictive value (NPV). An inter-reader reliability analysis using the kappa statistic was performed to determine consistency between two radiologists regarding the diagnosis of COVID-19. Cohen suggested the Kappa result be interpreted as follows: values ≤ 0 as indicating no agreement and 0.01–0.20 as none to slight, 0.21–0.40 as fair, 0.41–0.60 as moderate, 0.61–0.80 as substantial, and 0.81–1.00 as almost perfect agreement [15]. Bland–Altman and Passing–Bablok regression methods were used to describe the agreement between low-dose and ultra-low-dose CT scores [16]. Values less than 0.5 are indicative of poor agreement, values between 0.5 and 0.75 indicate moderate, values between 0.75 and 0.9 indicate good, and values greater than 0.90 indicate excellent agreement [17]. Analyses were performed using Statistical Package for Social Sciences (SPSS) program version 25.0.

### Ethics

Institutional review board approval was obtained.

## Results

A total of 167 [81, (48.5%) were male] patients were included. The mean (± SD) age of patients was 54 (± 18) years (range 19–89 years). The mean (± SD) body mass index (BMI) was 26.19 (± 4.63) kg/m^2^ (range 16.96–43.26). Thirty patients (18%) were obese (BMI ≥ 30).

**Fig. 1:**
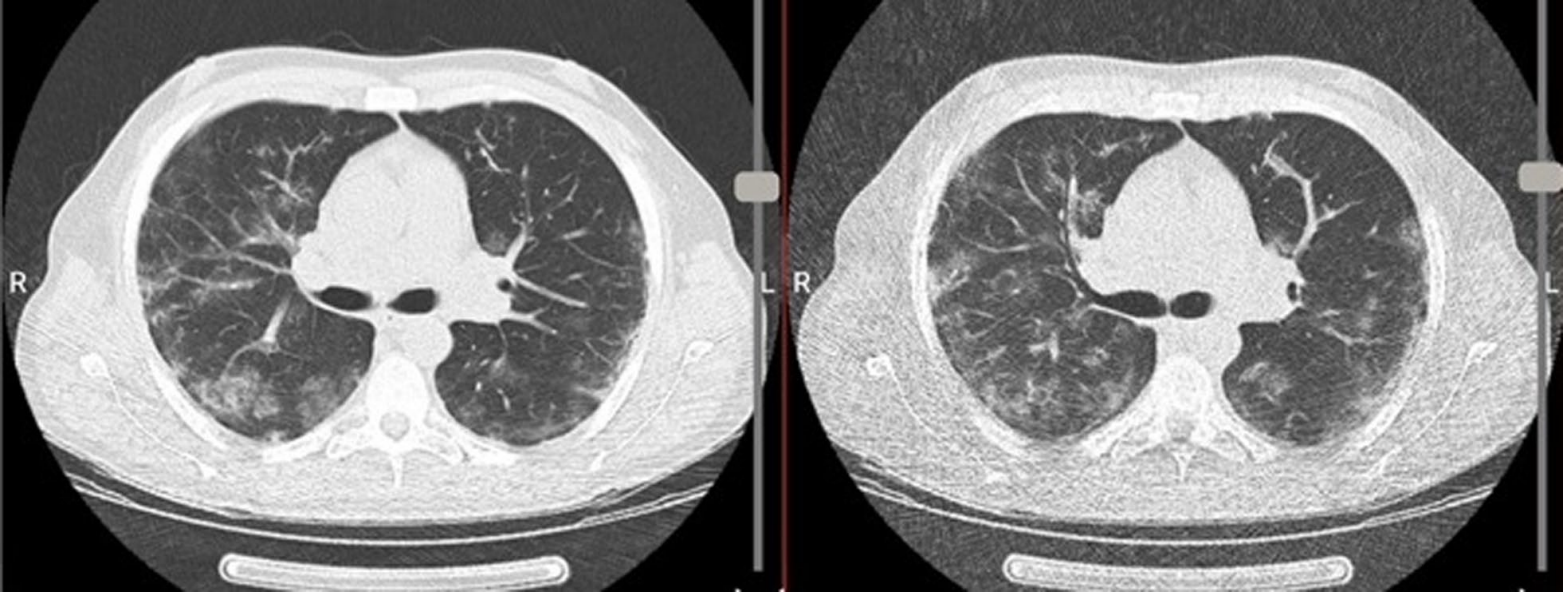
Bilateral multifocal subpleural and peribronchovascular ground-glass opacities in low-dose (left image) and ultra-low-dose (right image) CT scans typical for COVID-19 infection

**Fig. 2:**
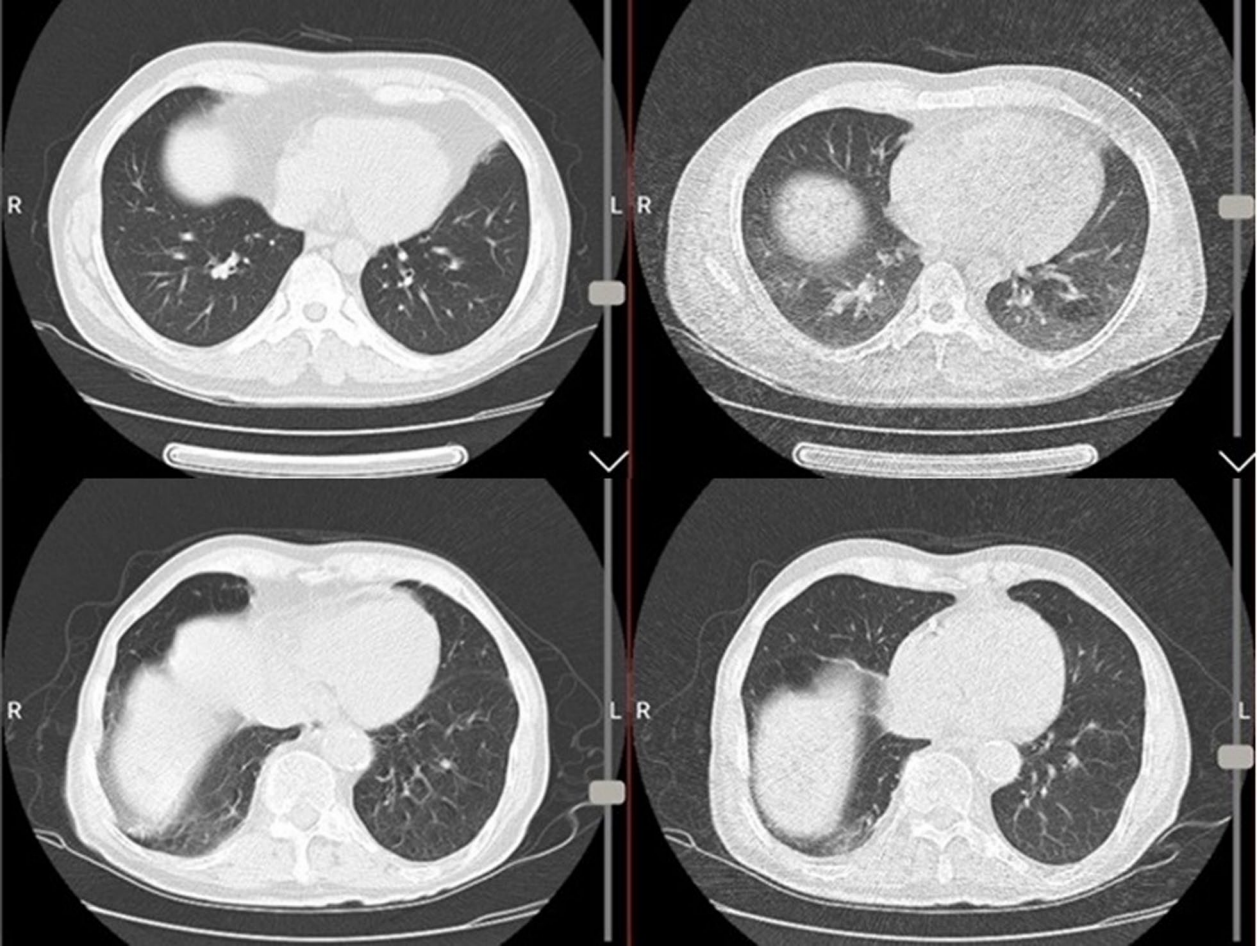
Examples of false positive read-out in ultra-low-dose protocol (upper row-right image) which was due to scan in expiratory-phase resulted in accentuated air-trapping and pseudo-ground glass appearance and was normal in same section of low-dose protocol (upper row-left image). Similar artifact is present in ultra-low-dose images of another case with false read-out of ground glass patch in right lung base (lower row-right image) which was mild atelectasis in low-dose section (lower row-left image)

On low-dose chest CT, 44 patients (26.3%) had findings consistent with COVID-19 infection and 121 patients (72.5%) without CT findings of COVID-19 infection. Ultra-low-dose chest CT had sensitivity and specificity values of 100% and 98.4%, respectively compared to low-dose chest CT as the reference, for imaging findings of COVID-19 pneumonia (Fig. 1). Two patients (1.2%) were falsely categorized to have pneumonia on ultra-low-dose CT scan (Fig. 2). Upon further review, these two false positive results were due to expiratory phase imaging that aggravated the lung markers and air-trapping at lung bases accentuated by increased noise in ultra-low-dose protocol at lung bases.

PPV and NPV of ultra-low-dose CT scan were respectively 95.7% and 100% and diagnostic accuracy of ultra-low-dose CT scan was 98.8% (95% confidence interval = 95.7–99.7%). There was considerable agreement between low-dose and ultra-low-dose methods (kappa = 0.97; *P* < 0.001); Table 2.

**Table 2:** Diagnostic accuracy value of ultra-low-dose CT scan findings compared to low-dose chest CT

	Low-dose chest CT						Diagnostic accuracy (95%CI)	Kappa (*P* value)
	Present	Absent	Sensitivity (95%CI)	Specificity (95%CI)	PPV (95%CI)	NPV (95%CI)		
*Ultra-low-dose chest CT*								
Present	44 (26.3)	2 (1.2)	100 (89.9–100)	98.4 (93.7–99.7)	95.7 (84–99.2)	100 (96.1–100)	98.8 (95.7–99.7)	0.83 (< 0.001)
Absent	0	121 (75.5)						

*NPV* negative predictive value, *PPV* positive predictive value, *CI* confidence interval

The inter-rater reliability for the two readers (SA and GI) was found to be kappa = 0.77 (*P* < 0.001) for ultra-low-dose and kappa = 0.81 (*P* < 0.001) for low-dose CT scan which was substantial and almost perfect, respectively.

**Fig. 3:**
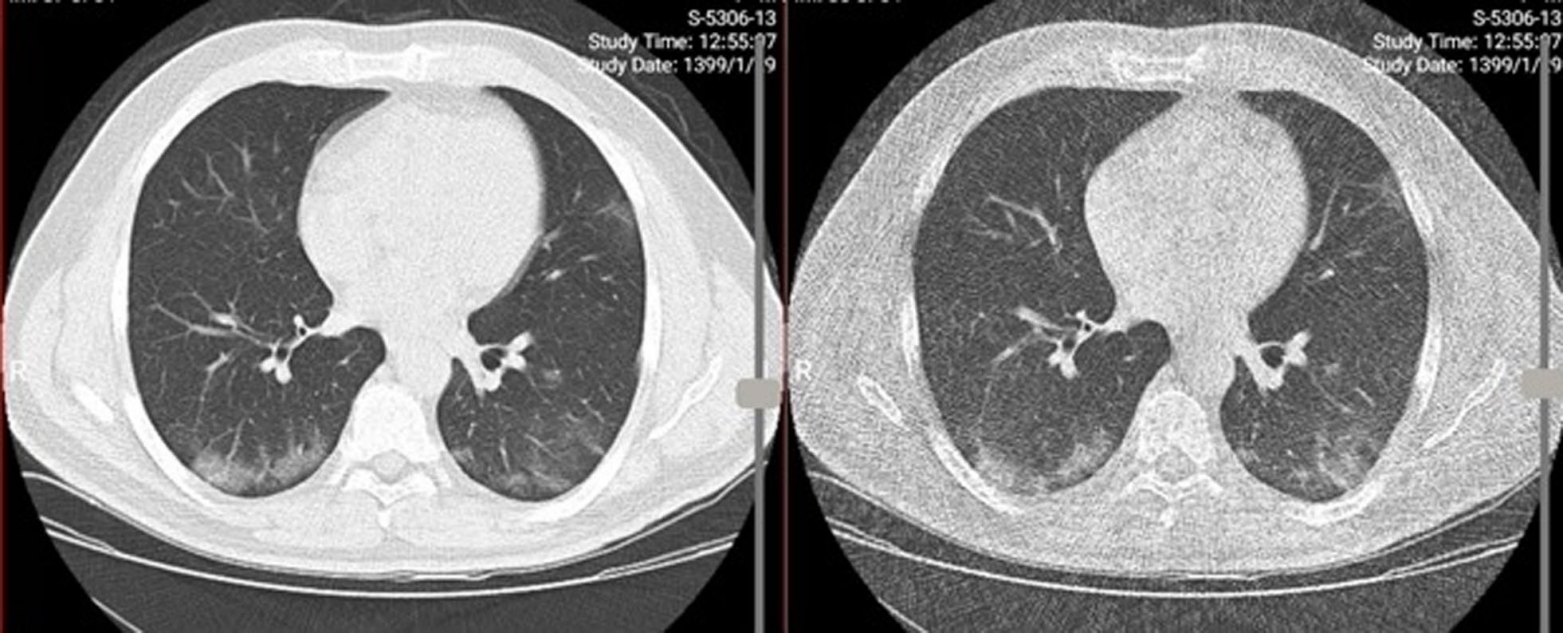
Suspected reverse halo in ultra-low-dose images (right image) not clearly seen in low-dose protocol (left image)

Table 3 presents the agreement between COVID-19 features on low-dose and ultra-low-dose chest CT scans. As seen, perfect agreement between low-dose and ultra-low-dose scans was found regarding diagnosis of ground-glass opacity, consolidation, reticulation, and nodular infiltration. None of the patients had reverse halo sign in low-dose protocol. However, a single reverse halo sign was reported on ultra-low-dose CT scan (Fig. 3). One patient had lymphadenopathy and four patients had pleural effusion on both low-dose and ultra-low-dose scans (kappa = 1, *P* < 0.001) with complete agreement.

**Table 3:** Diagnostic accuracy value of ultra-low-dose CT scan findings compared to Low-dose chest CT in COVID-19 features (*n* = 167)

Ultra-low-dose chest CT		Low-dose chest CT		Sensitivity	Specificity	PPV	NPV	Accuracy	Kappa (*P* value)
		Present	Absent						
Ground-glass opacity	Present	36 (21.6)	4 (2.4)	85.7	96.8	90.0	95.3	94.0	0.84 (< 0.001)
	Absent	6 (3.6)	121 (72.5)						
Crazy paving	Present	1 (0.6)	0 (0)	33.3	100	100	98.8	98.8	0.50 (< 0.001)
	Absent	2 (1.2)	164 (98.2)						
Consolidation	Present	23 (13.8)	2 (1.2)	88.5	98.6	93	97.9	97.0	0.88 (< 0.001)
	Absent	3 (1.8)	139 (83.2)						
Reticulation	Present	13 (7.8)	3 (1.8)	86.7	98.0	81.3	98.7	97.0	0.82 (< 0.001)
	Absent	2 (1.2)	149 (89.2)						
Nodular infiltration	Present	16 (88.9)	2 (11.1)	88.9	98.7	88.9	98.7	97.6	0.88 (< 0.001)
	Absent	2 (1.3)	147 (98.7)						

*NPV* negative predictive value, *PPV* positive predictive value

Table 4 shows distribution and laterality of the lesions on low-dose and ultra-low-dose chest CT scans. There were good agreements between low-dose and ultra-low-dose CT scans regarding distribution and laterality of the lesions.

**Table 4:** Distribution and laterality of the lesions on low-dose and ultra-low-dose chest CT scans among 167 patients

Ultra-low-dose chest CT	Low-dose chest CT				Kappa (*P* value)
Distribution	None	Peripheral	Central	Both	
None	113 (67.7)	1 (0.6)	0	1 (0.6)	0.86 (< 0.001)
Peripheral	4 (2.4)	17 (10.2)	0	4 (2.4)	
Central	0	0	3 (1.8)	1 (0.6)	
Both	0	0	0	23 (100)	
Laterality	None	Left-sided	Right-sided	Bilateral	
None	109 (65.3)	2 (1.2)	1 (0.6%)	0	0.90 (< 0.001)
Left-sided	1 (0.6)	16 (9.6)	0	0	
Right-sided	1 (0.6)	0	4 (2.4)	1 (0.6)	
Bilateral	2 (1.2)	0	0	30 (18.0)	

### Agreement between ultra-low and low dose CT score

**Fig. 4:**
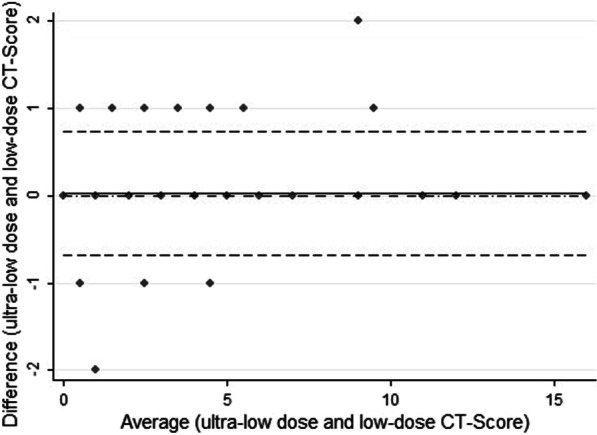
Bland–Altman plot of difference in CT score (ultra-low-dose score minus low-dose CT score) against the mean score of the two methods; cases over limit = 10 (5.99%); cases under limit = 6 (3.59%)

CT score ranged between 1 and 16 in both protocols with mean of 3.3 (± 3.3) in ultra-low-dose and 3.8 (± 3.4) in low-dose protocol. The Bland–Altman analysis results to assess agreement between two measurements of CT score (low-dose and ultra-low-dose) are displayed in Fig. 4. The mean difference between the two scores was 0.023 with the 95% agreement limits of − 0.031 to 0.079 and 16/167 = 9.5% of data outside the limits of agreement. Lin’s concordance correlation coefficient of absolute agreement was 0.99.

**Fig. 5:**
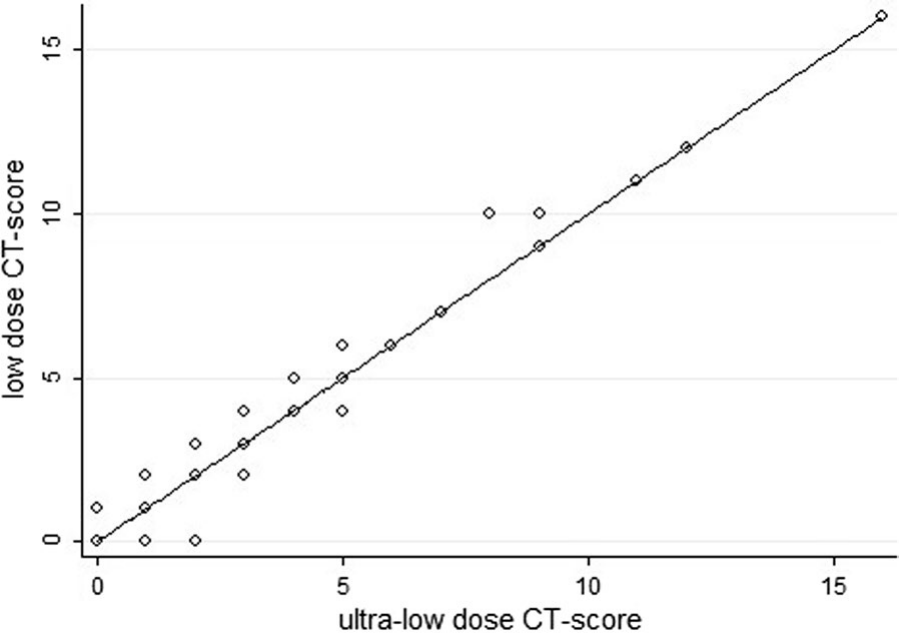
Passing–Bablok regression method to assess agreement between low-dose and ultra-low-dose of CT-score. Linearity Test (Test for deviation from linearity) had *P* > 0.20 and Passing-Bablok line was “*Y* = *X*”, with the *R*-square of 0.99

Passing-Bablok regression analysis results indicated that overall correlation of CT scan score measurements between low-dose and ultra-low dose was excellent and there was non-significant deviation from linearity in this association (*P* > 0.02); (Fig. 5). Pearson’s correlation coefficient was *r* = 0.99 (*P* < 0.001) for CT score between two protocols.

In ultra-low-dose protocol, radiation dose results including CTDI_vol_, DLP and ED were one third to one fourth less than similar numbers in low-dose protocol (Table 5).

**Table 5:** Radiation dose results in low-dose and ultra-low-dose protocols

	CTDI_vol_	DLP, Mean (± SD)	ED, Mean (± SD)	*P* value
Low-dose	1.15	36.5(± 5.2)	0.51(± 0.07)	< 0.001
Ultra-low-dose	0.34	9.9 (± 1.8)	0.14 (± 0.03)	

*CTDI*_*vol*_ CT dose index volume, *DLP* dose-length product, *ED* effective dose, *SD* standard deviation

### The effect of BMI

The sensitivity and specificity values of ultra-low-dose CT scan to diagnose consistent features of COVID-19 infection in patients with BMI values less than 30 kg/m^2^ were 100% and 97.3%. These values were respectively 100% and 85.7% in those whose BMI values were more than 30 kg/m^2^.

## Discussion

The aim of this study was to compare viral pneumonia findings between ultra-low-dose and low-dose chest CT scan during the COVID-19 pandemic. Since RT-PCR was not available, especially at earlier stages of the pandemic, lung CT scan had an integral role in the screening and diagnosis of COVID-19 pneumonia. A new screening and triage algorithm has been proposed by chest CT imaging of suspicious patients [18]. Many medical centers adhered to the proposed strategy, and chest CT imaging was used for the triage of suspected patients. This in turn is translated to an increasing pattern in the number of chest CT scans and consequently a higher radiation dose [19]. When this high number of CT acquisitions is coupled with repeated imaging for following progression/absorption of the lesions, patients are exposed to high degrees of irradiation. Therefore, keeping the radiation dose as low as possible became a priority for patient safety purposes. First, this goal was investigated using a low-dose chest CT approach (< 3 mGy) that has been recommended by some experts [20, 21]. The evidence shows that low-dose chest CT had high sensitivity (96.6%) for triage of COVID-19 in a study by Bahrami-Motlagh et al. [22] on 163 patients with suspected COVID-19 where 80 cases had positive RT-PCR results. At the next step, we intended to investigate ultra-low-dose chest CT and determine its sensitivity and specificity for triage purposes. Hence, we tried to assess the agreement between the two methods in diagnosing characteristics on chest CT suggestive of viral pneumonia during the COVID-19 pandemic.

New CT scanning machines with innovations in dose-sparing technologies have the benefit of lowering the radiation dose level. Effective radiation dose of ultra-low-dose can be as low as 0.14–0.5 mSv. This dose is very similar to the effective dose of a chest radiography (0.1 mSv) [6, 8]. This very low radiation dose with better image quality for visualization of abnormalities is advantageous to chest radiography. In case that diagnostic value of ultra-low-dose CT is satisfactory, this method can have significant clinical implications. In a study on a large sample of confirmed COVID-19 cases, Kuo et al. [23] reported that chest radiography had no role in screening of asymptomatic patients. A comparative study on 56 patients with mean age of 14 years and laboratory confirmed COVID-19 by Das et al. [24] showed that some suggestive abnormalities such as GGO and consolidation were detected in 46% of patients (26 cases) on chest CT. However, chest radiography detected the abnormalities in only 11 patients (19.6%). Therefore, a modality that can provide image quality similar to chest CT and superior to chest radiography, but with much lower radiation dose (i.e., ultra-low-dose chest CT) would be of interest for radiologists.

Our findings suggested that ultra-low-dose chest CT scan had a very good diagnostic performance for diagnosis of lung infiltration suggestive for viral pneumonia during the COVID-19 pandemic compared to low-dose CT scan with a sensitivity of 100% and specificity of 98.4%. Most abnormalities included GGO and consolidation. Overall, CT scores were low as the population studied comprised asymptomatic patients admitted for elective procedures. According to the Radiological Society of North America Expert Consensus Statement, GGO with peripheral distribution accompanied by consolidation is considered a typical appearance of COVID-19 pneumonia [13]. In a similar study to describe the diagnostic value of ultra-low-dose chest CT compared to standard dose CT, Greffier et al. [25] found that 97 patients out of 380 cases suspected to have viral pneumonia had CT patterns compatible with a viral pneumonia. Ultra-low-dose CT had a sensitivity of 98.9% and a specificity of 99% compared to standard dose CT as the reference standard to diagnose viral pneumonia pattern. Similar to our study, the patients were recruited during the COVID-19 outbreak between March and April 2020. Additionally, the CT patterns used to define viral pneumonia were those used by the current study including bilateral diffuse GGO, patchy consolidations, crazy paving, and other less frequent abnormalities. The mean effective radiation dose was 0.2 for ultra-low-dose and 1.6 for standard dose CT protocols.

Introducing imaging methods with lower radiation dose is promising for diagnosing COVID-19 pneumonia. Of course, a major concern in performing chest imaging, besides its accuracy, is radiation dose. Low-dose and ultra-low-dose CT scans are useful methods that can be implemented in such settings [26]. A previous study examined low-dose CT scan for this purpose with promising results [4] with sensitivity of 86% and specificity of 93%. According to our findings, ultra-low-dose chest CT was a reliable method with good accuracy when compared to low-dose chest CT in the diagnosis of COVID-19 pneumonia. Mean effective radiation dose in ultra-low-dose group was 0.14 mSv that is similar to the radiation dose of antero-posterior chest radiography of 0.14 mSv [27].

There was one false positive result in the ultra-low-dose group, detected as ground glass opacity at the lung bases, which was not confirmed on low-dose images. This finding was due to increased soft tissue thickness and noise at the lung base, which will be accentuated by inadequate inspiration and obesity. We suggest using a higher tube current of 20 mA instead of 10 mA in cases with high BMI to overcome this limitation.

Our study has several limitations. We were not able to perform RT-PCR to confirm COVID-19 infection in patients with suggestive imaging findings. This was due to a shortage of diagnostic kits at the time of the study conduction. However, this study focused on comparing the imaging abnormalities between ultra-low-dose and low-dose CT scans, not confirming the diagnosis of COVID-19. Our population study mainly consisted of asymptomatic candidates for elective surgeries during the COVID-19 outbreak, which resulted in fewer positive cases with less severe lung involvement that limits the generalizability of our results. A significant number of COVID-19 patients will require serial chest CT scans for different reasons. Unfortunately, follow-up ultra-low-dose scans were not obtained in our study; therefore, we were not able to evaluate its accuracy compared to the low-dose CT scan.

## Conclusion

Ultra-low-dose chest CT is an accurate method with less radiation compared to low-dose CT to diagnose lung infiltrations during the COVID-19 pandemic in patients admitted for elective or semi-urgent medical/surgical procedures. This technique could be used instead of low-dose CT during outbreaks when high number of patients may require chest imaging and there is shortage of diagnostic kits or there is uncertainty regarding the accuracy of laboratory tests. We suggest performing further studies to determine accuracy of ultra-low-dose chest CT in comparison to laboratory diagnosis and its role in the follow-up of COVID-19 patients.

## Data Availability

The data of the study can be provided for other researchers if sound request is sent to the corresponding author.

## Abbreviations

CT: Computed tomography
COVID-19: Coronavirus disease 2019
ED: Effective dose
DLP: Dose-length product
CTDIvol: CT dose index volume
PPV: Positive predictive value
NPV: Negative predictive value
